# Strong Tetrel Bonds: Theoretical Aspects and Experimental Evidence

**DOI:** 10.3390/molecules23102642

**Published:** 2018-10-15

**Authors:** Mehdi D. Esrafili, Parisasadat Mousavian

**Affiliations:** Laboratory of Theoretical Chemistry, Department of Chemistry, University of Maragheh, Maragheh 5513864596, Iran; p.mosavian1327@gmail.com

**Keywords:** noncovalent interaction, tetrel-bond, *σ*-hole, electrostatic potential, ab initio

## Abstract

In recent years, noncovalent interactions involving group-14 elements of the periodic table acting as a Lewis acid center (or tetrel-bonding interactions) have attracted considerable attention due to their potential applications in supramolecular chemistry, material science and so on. The aim of the present study is to characterize the geometry, strength and bonding properties of strong tetrel-bond interactions in some charge-assisted tetrel-bonded complexes. Ab initio calculations are performed, and the results are supported by the quantum theory of atoms in molecules (QTAIM) and natural bond orbital (NBO) approaches. The interaction energies of the anionic tetrel-bonded complexes formed between XF_3_M molecule (X=F, CN; M=Si, Ge and Sn) and A^−^ anions (A^−^=F^−^, Cl^−^, Br^−^, CN^−^, NC^−^ and N_3_^−^) vary between −16.35 and −96.30 kcal/mol. The M atom in these complexes is generally characterized by pentavalency, i.e., is hypervalent. Moreover, the QTAIM analysis confirms that the anionic tetrel-bonding interaction in these systems could be classified as a strong interaction with some covalent character. On the other hand, it is found that the tetrel-bond interactions in cationic tetrel-bonded [p-NH_3_(C_6_H_4_)MH_3_]^+^···Z and [p-NH_3_(C_6_F_4_)MH_3_]^+^···Z complexes (M=Si, Ge, Sn and Z=NH_3_, NH_2_CH_3_, NH_2_OH and NH_2_NH_2_) are characterized by a strong orbital interaction between the filled lone-pair orbital of the Lewis base and empty BD*_M-C_ orbital of the Lewis base. The substitution of the F atoms in the benzene ring provides a strong orbital interaction, and hence improved tetrel-bond interaction. For all charge-assisted tetrel-bonded complexes, it is seen that the formation of tetrel-bond interaction is accompanied bysignificant electron density redistribution over the interacting subunits. Finally, we provide some experimental evidence for the existence of such charge-assisted tetrel-bond interactions in crystalline phase.

## 1. Introduction

Over the past decades, there has beenan increasing awareness of the importance of noncovalent interactions owingto their critical roles in various fields of chemistry and biochemistry, such as protein folding, molecular recognition, drug design and crystal packing [1,2,3]. Of the various noncovalent interactions, hydrogen-bonding (H-bonding) has emerged as the most extensively studied case [4,5,6,7,8]. It is typically formulated as an attractive Lewis acid-Lewis base interaction, D-H···A, between the electron-deficient hydrogen atom of one molecule (D-H), acting as a bridge to an electron-rich site on the other molecule (A). However, much attention has been recently devoted to other types of noncovalent interactions like *σ*-hole bonding due to their useful applications in supramolecular chemistry, crystal engineering, and biochemistry [9,10,11,12,13,14,15,16,17]. A *σ*-hole bond [18,19,20,21,22,23,24] is a noncovalent interaction analogous to the H-bonding, in which a covalently bonded atom of groups 14–18 of the periodic table, rather than an H atom, serves a similar function as a bridge between two molecules. For example, the possibility of noncovalent interaction between some halocarbons and potential Lewis bases has been known for some time [25,26] and has continued to be studied at a rapidly increasing rate in recent years [27,28,29,30]. It has been found that the halogen atom in these molecules is able to develop a positive *σ*-hole region on the outermost portion of the halogen atom, along the C–X atoms (X=F, Cl, Br, I). The emergence of such a positive area, which may seem surprising due to the high electronegativity of halogen atoms, is responsible for the high directionality and formation of an electrostatically driven interaction with a negative region on the Lewis base. Note also that besides these electrostatic effects, there are also polarization effects and a substantial charge-transfer from the Lewis base into the BD*_C-X_ antibonding orbital [31,32,33], precisely analogous to the case of a H-bond. The *σ*-hole interaction involving the halogen atoms is also known as halogen-bonding in view of the concept of H-bonding. Furthermore, it is not only the halogen atoms which can act as a Lewis acid center, but the elements of groups 14, 15, 16 and 18 of the periodic table as well, in which the resulting *σ*-hole interaction is called a tetrel-bonding [34,35,36,37,38,39], pnicogen-bonding [40,41,42,43,44], chalcogen-bonding [45,46,47,48,49] and aerogen-bonding [50,51,52,53], respectively.

Generally, *σ*-hole interactions share many common physical and chemical properties with the more traditional H-bonding. They offer a rich array of possibilities to design and fabricate new materials with desired properties, in areas ranging from pharmaceuticals to crystal growth [54,55,56,57]. Such diverse applications of *σ*-hole interactions mainly originate from their directional tunability. For example, the strength and properties of tetrel-bonds can be tuned not only by changing the tetrel atom (group 14 elements) itself, but also by changing the electron withdrawing/accepting ability of the reminder of the molecule [37,58,59,60,61]. As a result, a broad range of interaction energies may be spanned by changing the C atom in the tetrel-bond donor into a Si, Ge or Sn atom. Meanwhile, the Lewis base moiety in the tetrel-bond interaction could vary from anions like F^−^ or Cl^−^ [34,62,63], through lone-pair electrons on nitrogen or oxygen [64,65,66], to π electrons in unsaturated bonds [67,68]. The latter may offer a further opportunity to tune the strength of tetrel-bonds and therefore expand their application scope [69,70].

A series of systematic experimental and theoretical studies have produced detailed descriptions of tetrel-bonds in either crystalline state or gas phase. However, these studies have mostly focused on the neutral complexes. For example, Mitzel et al. [71] have found short Si···N contacts in the crystalline structure of Si(ONMe_2_)_4_ and related compounds. Thomas and coworkers have provided an experimental evidence for the tetrel-bonding in crystalline structures based on charge density analysis. Alkorta and coworkers have investigated tetrel-bonding interactions between SiXY_3_ (X and Y=H, F, and Cl) and some electron-rich groups (NH_3_, NCH, CNH, OH_2_, and FH) [72]. A detailed computational study by Mani and Arunan [73,74] has also found unusual tetrel-bond interactions called “carbon bonding” in the complexes of methanol as the tetrelbond donor with different Lewis bases. The formation of the latter interactions has also been proposed as a preliminary stage of the SN2 reaction by Grabowski [75]. These studies clearly showed that tetrel-bonding is moderately strong and could act as a possible molecular linker in crystal engineering and supramolecular chemistry, similar to H-bonding.

**Scheme 1:**
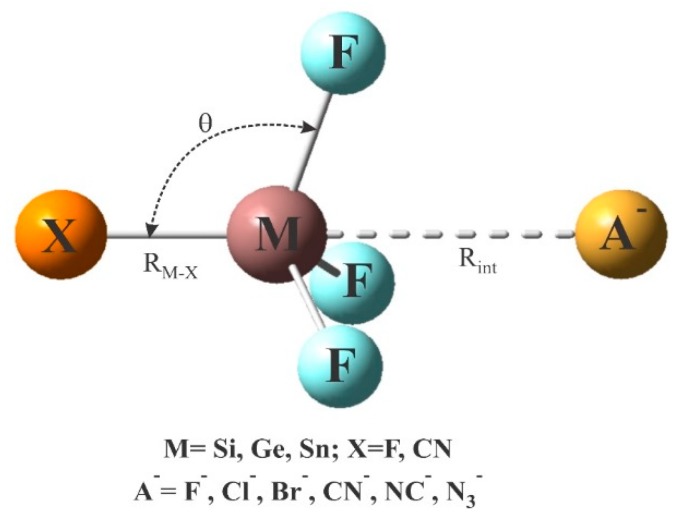
Representative geometrical structure of anionic tetrel-bonded complexes.

**Scheme 2:**
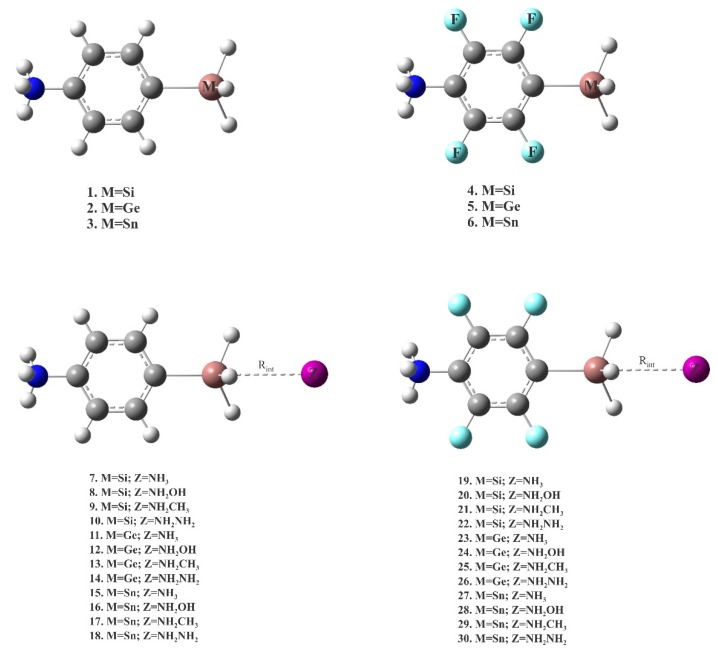
Structure of monomers **1**–**6** and cationic tetrel-bonded complexes **7**–**30**.

Recently, Scheiner has reported [76] a detailed study on the ability of hydrogen, halogen, chalcogen, pnicogen, and tetrel-bonds as a potential halide (F^−^, Cl^−^, Br^−^) receptor. It was found that the tetrel-bonding exhibits a quite larger tendency to bind to halides than other *σ*-hole interactions. In another study [77], the author has also shown that the addition of a -SnF_3_ group to either an imidazolium or triazolium ion provides a strong halide receptor. Interestingly, the tetrel-bonding receptors bind far more strongly to each anion than an equivalent number of K^+^ counterions. In the present study, we perform a systematic study on the strength and characteristic of charge-assisted tetrel-bond interactions in some model complexes (Scheme 1 and Scheme 2). The nature of anionic as well as cationic tetrel-bonds is analyzed by means of molecular electrostatic potential (MEP), quantum theory of atoms in molecules (QTAIM), natural bond orbital (NBO) and electron density difference (EDD) methods. The influence of different substituents on either Lewis acid or Lewis base is also studied in detail. Moreover, the characteristics of these charge-assisted complexes are compared with those of available neutral ones. Finally, we provide some experimental evidence for the existence of such charge-assisted tetrel-bonds in crystalline structures and supramolecular assemblies.

## 2. Systems and Methods

In this work, we report the results of ab initio calculations to study charge-assisted tetrel-bond interactions for two different sets of model systems. In the first model, XF_3_M molecule (X=F, CN; M=Si, Ge and Sn) interacts with A^−^ anions (A^−^=F^−^, Cl^−^, Br^−^, CN^−^, NC^−^ and N_3_^−^). This allows us to check the possibility of anionic tetrel-bonding interaction in the mentioned complexes. The second model studied here includes the cationic tetrel-bonded [p-NH_3_(C_6_H_4_)MH_3_]^+^···Z complexes, in which M=Si, Ge, Sn and Z=NH_3_, NH_2_CH_3_, NH_2_OH and N_2_H_4_. The H atoms of the benzene ring are additionally substituted by F atoms in order to study substituent effects.

All ab initio calculations were performed using the Gaussian 09 package [78]. The MP2 method was used, along with the aug-cc-pVTZ basis set to optimize geometries of the anionic XF_3_M:A^−^ tetrel-bonded complexes. Frequency calculations were performed at the same computational level to ensure that the optimized structures correspond a true minimum on the potential energy surface. In the case of the cationic tetrel-bonded systems, the geometry optimizations and the corresponding frequency calculations were performed at the MP2/aug-cc-pVDZ level. Single-point calculations with a larger aug-cc-pVTZ basis set were then performed using the aug-cc-pVDZ optimized geometries. The interaction energies for both sets of the complexes were computed at the MP2/aug-cc-pVTZ level, as the difference between the energy of the complex and the energy sum of the isolated monomers, and corrected for the basis set superposition error (BSSE) by using the Boys–Bernardi counterpoise method [79].

To evaluate the possible orbital interactions between the interacting monomers, the NBO analysis was performed with the NBO 5.0 program (Theoretical Chemistry Institute, University of Wisconsin, Madison, Wisconsin, United States) [80]. The most positive (V_S,max_) and most negative (V_S,min_) electrostatic potentials of the isolated monomers were obtained using the Wave Function Analysis-Surface Analysis Suite (WFA-SAS) [81]. The QTAIM analysis was performed by means of the AIM2000 program [82] with the MP2/aug-cc-pVTZ generated wave functions. To see the amount of electron density shift due to the complex formation, the EDD isosurfaces were computed with the help of MultiWFN [83]. These were obtained by subtracting the electron density of the complex with the sum of the electron densities of the interacting monomers with the geometries in the optimized complex.

## 3. Results and Discussion

### 3.1. Anionic Tetrel-Bonds

**Figure 1:**
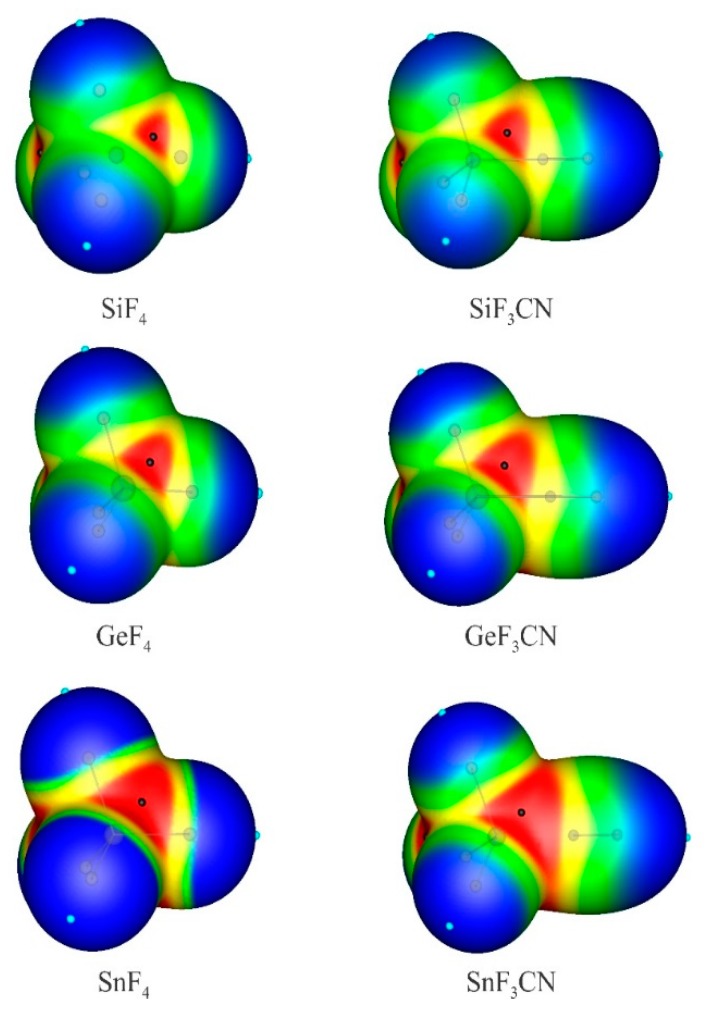
MEPs of isolated monomers. The color code, in kcal/mol, is: red > 40; 40 > yellow > 20; 20 > green > 0 and blue < 0. The small black and blue circles indicate surface maxima and minima, respectively.

Scheme 1 indicates the general structure of anionic tetrel-bonded complexes XF_3_M:A^−^, in which the M atom of XF_3_M acts as the Lewis acid site to interact with the excess electron density over the anions A^−^. It should be mentioned that although there might be many minima on the potential energy surface of these complexes, we are interested here in the interaction involving the linear X-M···A^−^ arrangement. The corresponding optimized geometries at the MP2/aug-cc-pVTZ level are summarized in Figure S1 of Supporting Information. All these complexes are found to have a favorable X−M···A^−^ linear arrangement. The binding distances and interaction energies of these complexes are listed in Table 1. According to the previous studies [37,62,65,67,84,85], the formation of such anionic tetrel-bonding interactions can be largely attributed to the localization of a positive electrostatic potential over the M atom, in the extension of the M-X bond. Indeed, the MEP analysis of XF_3_M monomers in Figure 1 reveals that the maximum positive electrostatic (V_S,max_) of the Sn atom (96.5 kcal/mol) in SnF_4_ is greater than that of Ge (70.2 kcal/mol) and Si (57.3 kcal/mol) in GeF_4_ and SiF_4_, respectively. In the case of MF_3_CN monomers, it is seen that the *σ*-hole potential associated with the M atom becomes more positive as the size of the M atom increases. Consequently, it is expected that XF_3_M molecules can participate in a *σ*-hole interaction with the *σ*-hole acting as a Lewis acid center, and the strongest acidic properties are predicted for the Sn atom of SnF_4_ and SnF_3_CN based on the MEP analysis.

As Table 1 indicates, the M···A^−^ binding distances of XF_3_M:A^−^ complexes are in the range of 1.662–2.523 Å, which are much shorter than the sum of the van der Waals (vdW) radii of the interacting atoms [86]. This clearly shows the existence of a strong interaction between the XF_3_M and A^-^ moieties. In many cases, the M atom is characterized by pentavalency, i.e., is hypervalent. This is similar to the one described for the transition state structure of SN2 reaction between a tetrel atom center and anion species [75,87]. In fact, most of the M···A^−^ binding distances are short enough to be considered covalent bonds which have lost some degree of covalency. The binding distances for a given anion increase in the order SiF_3_CN < SiF_4_ < GeF_3_CN < GeF_4_ < SnF_3_CN < SnF_4_. Note also that M···A^−^ distances become longer in the order of Si < Ge < Sn when the anion is the same, which is similar to the order of vdW or the covalent radius of these atoms (Si < Ge < Sn). The interaction between the anion and M atom is also able to induce a large distortion in the XF_3_M molecule, as evidenced by the calculated X-M-F angles (Table 1). For each set of the complexes, one can see that the X-M-F angles are close to 90°, which may provide further evidence for the strong interaction between the XF_3_M and A^−^ moieties.

**Figure 2:**
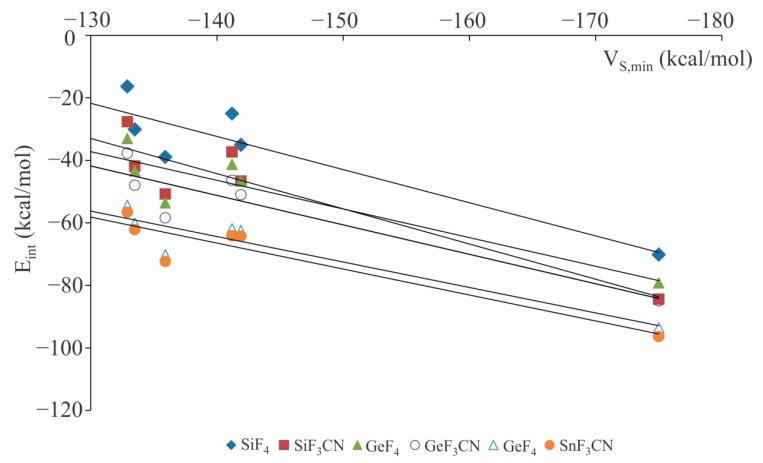
Correlation between the interaction energies and V_S,min_ values associated with the A^−^ anions in the binary XF_3_M:A^−^ complexes. The squared correlation coefficient (R^2^) value is 0.833, 0.831, 0.846, 0.839, 0.868 and 0.872 for SiF_4_, SiF_3_CN, GeF_4_, GeF_3_CN, SnF_4_ and SnF_3_CN complexes, respectively.

From Table 1, one can see that the interaction energies of XF_3_M:A^−^ are very large and negative, indicating a strong interaction between XF_3_M and A^−^ subunits. These results are in agreement with recent reports that indicate that tetrel-bonding can be used as a vehicle for strong and selective anion binding [77,88]. Moreover, the interaction energies obtained here are in good agreement with those of other related studies [62,63,76,77,88]. Comparing interaction energies clearly indicates that for a given XF_3_M, the value of interaction energy for F^−^ is systematically larger than other anions. In fact, such large negative interaction energies together with the corresponding very short binding distances indicate that the M···F^−^ interactions are mainly covalent in nature. Interacting with the same anion, SnF_4_ tends to form stronger tetrel-bond interaction than other molecules, as characterized by a larger interaction energies in the corresponding complexes. This has been found for other tetrel-bonds, previously [37,58,75,84,89]; the increase of the interaction energy for the analogue complexes if the atomic number of the tetrel atom increases. This is connected with the electrostatic nature of these interactions due to the presence of a large positive electrostatic potential on the central atom of XF_3_M molecules (Figure 1). Moreover, it is natural that the more negative electrostatic potential (V_S,min_) associated with the anion forms a more stable M···A^−^ interaction. However, as Figure 2 indicates, we found almost a poor linear correlation between the interaction energies of these complexes and V_S,min_ values associated with the anions. Note that such a poor linear relationship between the E_int_ and V_S,min_ values has already been described in the literature [90,91,92]. This is mainly related to the different nature of the A^−^ moiety in these complexes, which provides a distinct contribution of other energy terms such as polarization or charge-transfer in these systems.

The results of Table 1 also indicate that due to the formation of XF_3_M:A^−^ complexes, the M-X bonds are elongated. The magnitude of this bond elongation is in the range of 0.083–0.118 Å, 0.048–0.145 Å and 0.072–0.091 Å in the Si, Ge and Sn complexes, respectively. Note that the strongest M∙∙∙F^−^ interaction in these systems is characterized by a large elongation of M-X bond, which is much larger than the corresponding values in the Cl^−^ or Br^−^ complexes. Also, paired with the same anion, MF_4_ complexes show a relatively smaller variation in the M–X bond distances than MF_3_X analogues, which is based on the fact that the F is a poor leaving group than the CN. This result is consistent with the variation of interaction energy of these complexes, and suggests that Sn-X bond displays a larger red shift in the corresponding M-X stretching frequency than the Ge-X and Si-X ones. We will come back to this conclusion further on in our discussion when NBO analysis is illustrated.

**Figure 3:**
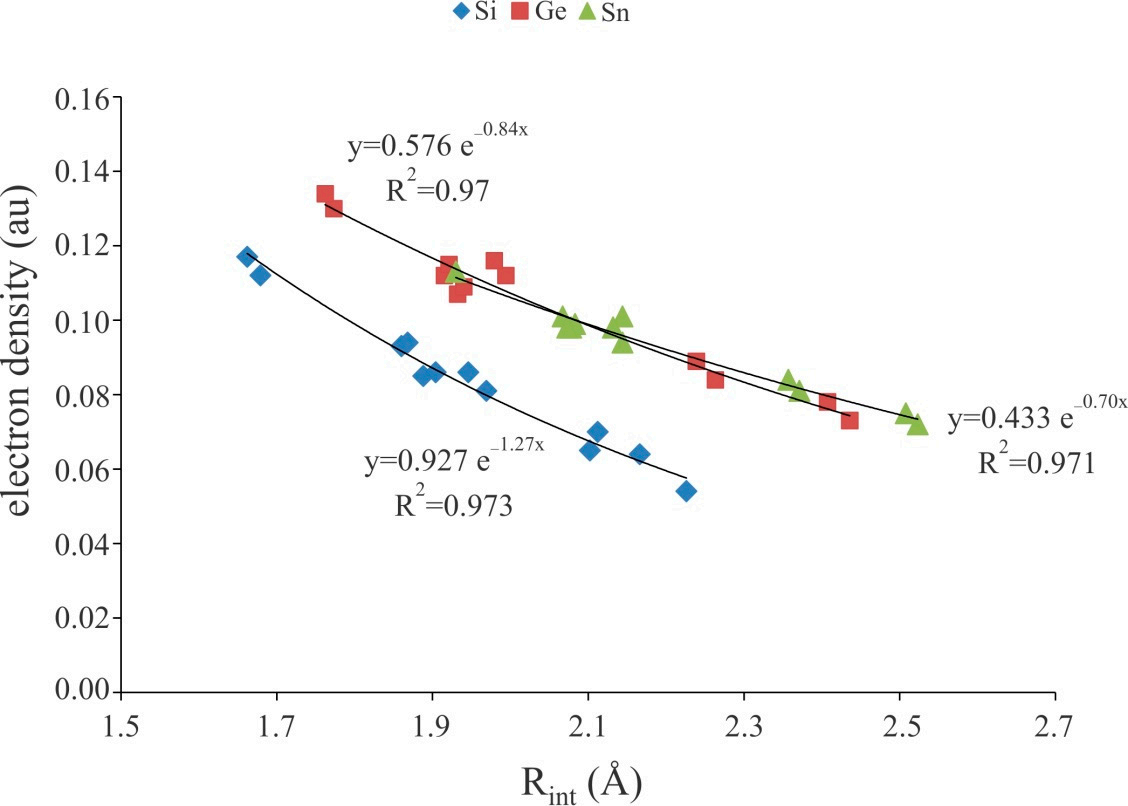
Exponential relationship between theM∙∙∙A^−^ binding distances and electron density at the corresponding BCPs of the XF_3_M:A^−^ complexes.

To have a deeper understanding of the nature of anionic tetrel-bond interactions, we have performed the topological analysis of the electron density of XF_3_M:A^−^ complexes (Table 2). It is found that for each system considered, there exists a bond critical point (BCP) associated with the M∙∙∙A^−^ interaction. As seen, the strong tetrel-bond interactions in XF_3_M:A^−^ complexes are characterized by a large electron density value at the corresponding bond critical points (BCPs), which are much larger than those of at the neutral tetrel-bonded systems [38,59,87,93,94]. For a given M or X, the F^−^ complexes exhibit the largest *ρ*_BCP_ value, while the smallest one corresponds to the Br^-^ complexes. Note also that, as predicted by the *ρ*_BCP_ values, the tetrel-bond interactions of the Sn complexes are stronger than those of Ge or Si ones. This is in line with the other related studies [65,72,75,95], where it was found that the *ρ*_BCP_ value is a good descriptor of the strength of interaction. Besides, almost a good exponential correlation was found between the binding distances and electron density values at the corresponding BCPs of XF_3_M:A^−^ complexes (Figure 3). Moreover, the Laplacian values of *ρ*_BCP_ are found to be positive and in the range of 0.053–0.936 au, which is indicative of closed-shell nature of these interactions [96]. Meanwhile, the negative values of total electron energy density at M∙∙∙A^−^ BCPs, H_BCP_, for all these complexes clearly confirm that the anionic tetrel-bond interactions could be classified as the strong interactions with some covalent character [97].

As noted earlier, charge-transfer from the electron donor into the empty orbital of the electron acceptor also plays an important role in the formation and stabilization of tetrel-bonded complexes [61,62,63,85,87,98]. For the anionic tetrel-bonded complexes studied here, it is expected that there exists a stabilizing orbital-orbital interaction between the lone-pair orbital of the anion, LP (A^−^), and empty anti-bonding M-X orbital of XF_3_M molecule (BD*_M-X_). The latter orbital interaction should be responsible for the elongation of M-X bonds and their red-shift upon the complexation. To confirm this, we performed NBO analysis on the XF_3_M:A^−^ complexes. Table 2 summarizes the calculated stabilization energy E^(2)^ values due to the LP (A^−^)→BD*_M-X_ orbital interaction. As is evident, these E^(2)^ values are quite large, especially for the A^−^=CN^−^ and F^−^ complexes, which demonstrates the significant role of the mentioned orbital interaction in these systems. It is also found that for all complexes analyzed here, the formation of tetrel bonds results in an increase in the positive charge of the M atom due to its polarization in the presence of the negative charge of the anion (Table 2). For each set of the complexes, such polarization is largest in the F^−^ complexes, which is consistent with the stronger tetrel-bond interaction in these systems. However, due to the variety of Lewis bases, it is not possible to find any regularity in the changes of the atomic charges here. The data in Table 2 also reveal that the net charge-transfer values (q_CT_) for the XF_3_M:A^−^ complexes are very large, with values ranging from 0.21 to 0.58 e. Moreover, for a given M or X, the N_3_^−^ and Br^−^ complexes are identified by a larger q_CT_ values compared to other ones, which is most likely due to the large polarizability of these moieties. Hence, the larger elongation of the M-X bond in the latter complexes can be attributed to the more favorable charge-transfer, which results in the partial population of the antibonding BD*_M-X_ orbital of XF_3_M and its redshift. As also shown in Table 2, the Wiberg bond index (WBI) of the anionic tetrel-bonds is large, which verifies the formation of covalent M∙∙∙A^−^ interactions in these systems.

**Figure 4:**
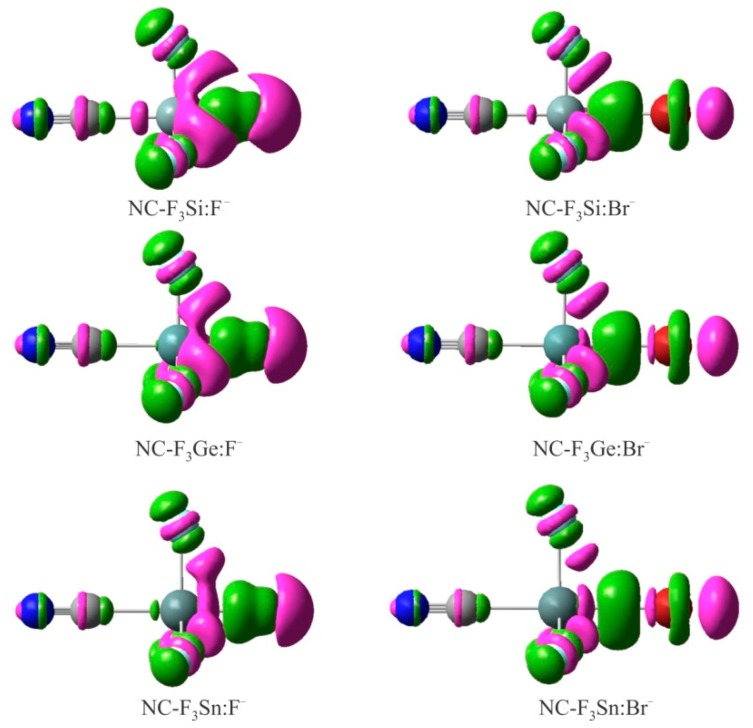
EDD isosurfaces (±0.005 au) of some representative XF_3_M:A^−^ complexes. The violet and green regions indicate regions of decreased and increased electron densities, respectively.

When the XF_3_M molecule is paired with the A^−^ anion, there is a mutual polarization between the two moieties, which can be verified using the EDD analysis. Figure 4 shows the EDD isosurfaces for some representative complexes of XF_3_M:A^−^, which were obtained by subtracting the electron density of the complex with the sum of the electron densities of the interacting monomers with the geometries in the optimized complex. Here, violet regions show a decreased electron density, while green areas refer to an increased electron density. As can be seen, the formation of these complexes leads to the appearance of a large electron density loss region over the M atom, facing the anion. The size of this electron density loss region becomes larger as the size of the M atom increases. Meanwhile, a large electron density accumulation is found between the M and A^−^, which confirms the formation of a covalent M∙∙∙A^−^ interaction in these systems. Moreover, the formation of anionic tetrel-bond interaction in these complexes tends to induce an accumulation of electron density on the F atom of XF_3_M. One can also see the localization of a large electron density loss region over the anion, which is related to the polarization of these moieties in the presence of positive σ-hole on the M atom. Clearly, such electron density shift is larger for the Sn complexes than Ge and Si ones, due to more positive *σ*-hole potential associated with the former systems.

### 3.2. Cationic Tetrel-Bonds

Scheme 2 depicts the general representation of cationic tetrel-bonded complexes **7**–**30** studied here. The corresponding optimized geometries are summarized in Figure S2 of the Supporting Information. Their intermolecular M···N distances and interaction energies are reported in Table 3. From Figure S2, one can see that all these complexes are characterized by a linear C-M···N interaction, in which nitrogen atom of the Lewis base is pointed towards the M atom of the Lewis acid. The binding distances are in the range of 2.175–2.567, 2.352–2.790 and 2.479–2.735 for the M=Si, Ge and Sn, respectively. All these binding distances are smaller than the sum of vdW radii of the respective atoms [86], which implies that there is an attractive interaction between the interacting molecules. For a given M, NH_2_NH_2_ forms always the shortest tetrel-bond distance, while the longest corresponds to NH_3_. Moreover, the substitution of F atoms in the benzene ring tends to decrease the binding distances, which can be attributed to the increase of positive electrostatic potential on the M atom due to presence of the F atoms. This indicates that the formation of cationic tetrel-bond in these systems is, at least partly, a consequence of the electrostatic attraction between the nitrogen atom of Lewis bases and the M atom. As also expected, the M···N binding distances for a fixed nitrogen base increase in the order of Ge > Sn > Si can be related to the combination result of the interaction energy and the atomic radius of these atoms. The results of Table 3 also show that the formation of cationic tetrel-bonds in the binary complexes **7**–**30** leads to a significant increase in the C-M-H angles (θ), as evidenced by θ values close to 90°. This is in line with previous studies, where it was found for the strong tetrel-bonded complexes that the intermolecular interaction should be a preliminary stage of the SN2 reaction [75,87].

Considering the interaction energies in Table 3, it is found that the most strongly bound complex **30** has an interaction energy of −26.98 kcal/mol, while the most weakly bound complex **7** has an interaction energy of only −7.69 kcal/mol. These interaction energies are larger than the reported values for similar tetrel-bond interactions in the related neutral complexes [65,93]. Meanwhile, the calculated interaction energies for the Si and Ge complexes are close to those of tetrel-bonding interactions in the prorogated complexes of pyridine-MF_3_ or furan-MF_3_ with NH_3_ [66]. Note that, for the same electron acceptor moiety, NH_2_NH_2_ tends to form more stable tetrel-bond interaction than others, which is not consistent with the V_S,min_value (in kcal/mol) associated with the nitrogen atom in thee bases: NH_3_(−42.7) > NH_2_CH_3_ (−38.6) > NH_2_NH_2_ (−36.5) > NH_2_OH (−28.8). This may be attributed in part to secondary interactions between these Lewis bases and the H atoms of -MH_3_ moiety in the Lewis acid. Moreover, this can be explained in the manner of the negative hyperconjugation effect on the side of Lewis bases as suggested by Zierkiewicz and Michalczyk [92]. This finding clearly reveals that the V_S,min_ value, a property in a single special point of the Lewis base, cannot be regarded as a good indicator of the cationic tetrel-bond interactions, and in addition to electrostatic effects other factors such as the polarization should play an important role in the stability and formation of these interactions.

**Figure 5:**
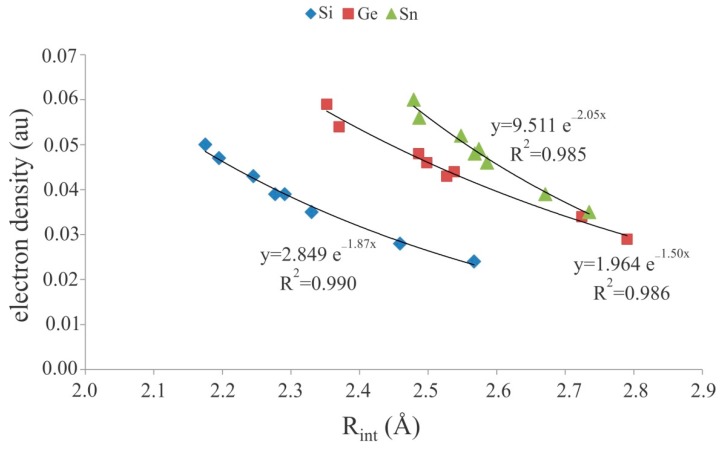
Exponential relationship between the M···N binding distances and electron density at the corresponding BCPs of the cationic tetrel-bonded complexes.

The topological analysis of the electron density of the complexes **7**–**30** exhibits the presence of a BCP between the M atom of Lewis acid and N atom of the nitrogen bases. The electron densities at the M···N BCPs are between 0.024 au in **7** and 0.060 au in **30** (Table 4). It is interesting to note that, like weak tetrel-bond interactions in neutral complexes [38,59,87,93,94], we found an exponential correlation between the electron density at the BCP and the interatomic distances of these systems (Figure 5). Importantly, the calculated squared correlation confection values (R^2^) for these cationic terel-bonded complexes are larger than those of anionic ones (Figure 3). Moreover, the positive ∇^2^*ρ*_BCP_ values associated with these complexes demonstrate that the cationic tetrel-bond interactions are within the closed-shell interaction regime. Meanwhile, negative total energy densities (H_BCP_) are obtained for all complexes studied here, which confirm that the cationic tetrel-bonds have some covalent character. Note that the for each set of the complexes, most negative values of H_BCP_ correspond to the stronger interactions and to the greater values of *ρ*_BCP_ (Table 4).

According to the NBO analysis, there is a noticeable charge-transfer interaction between the lone-pair bonding orbital LP(N) of nitrogen atom of the Lewis bases and the BD*_M-C_ antibonding orbital of the Lewis acid. A similar interaction between the lone-pair of nitrogen and the BD*_M-C_ anti-bonding orbital (M=C, Si, Ge) was analyzed recently for the prorogated complexes of pyridine-MF_3_ with NH_3_ [66]. This orbital interaction is responsible for a negligible elongation of the M-C bond in these complexes. The stabilization energies E^(2)^ associated with the latter orbital interaction are in the range of 9.22–18.60, 12.28–20.20 and 15.25–22.50 kcal/mol for the Si, Ge and Sn complexes, respectively. There is an almost linear correlation between these E^(2)^ values and interaction energies of these complexes (see Figure S3, Supporting Information), which demonstrates that the charge-transfer interaction also plays an important role in the stability of these systems. Note also that the F-substituted complexes **19**–**30** are characterized by quite a large E^(2)^ value with respect to the **7**–**18**. We note that in addition to the LP(N) → BD*_M-C_ orbital interaction, there is also some weak orbital interactions between the BD orbital of M-H to the BD*_O-H_, BD*_C-H_ or BD*_N-H_ antibonding orbital of NH_2_OH, NH_2_CH_3_ or NH_2_NH_2_ with the stabilization energies in the range of 1.20–6.85 kcal/mol. The calculated NBO charges also show a significant net charge transfer (q_CT_) in these complexes with values ranging from 0.072 e in **7** and 0.098 e in **30**. As expected, relatively larger q_CT_ values are found for the Sn complexes, which indicates that there exists a relationship between the size of transferred charge between the interacting monomers and interaction energy. This is evident in Figure S4 of the Supporting Information, where a linear correlation is seen between these quantities. Additionally, the data in Table 4 shows that the obtained WBI values for these complexes vary from 0.114 to 0.225, which suggests that these cationic tetrel-bonds have a considerable covalent character.

**Figure 6:**
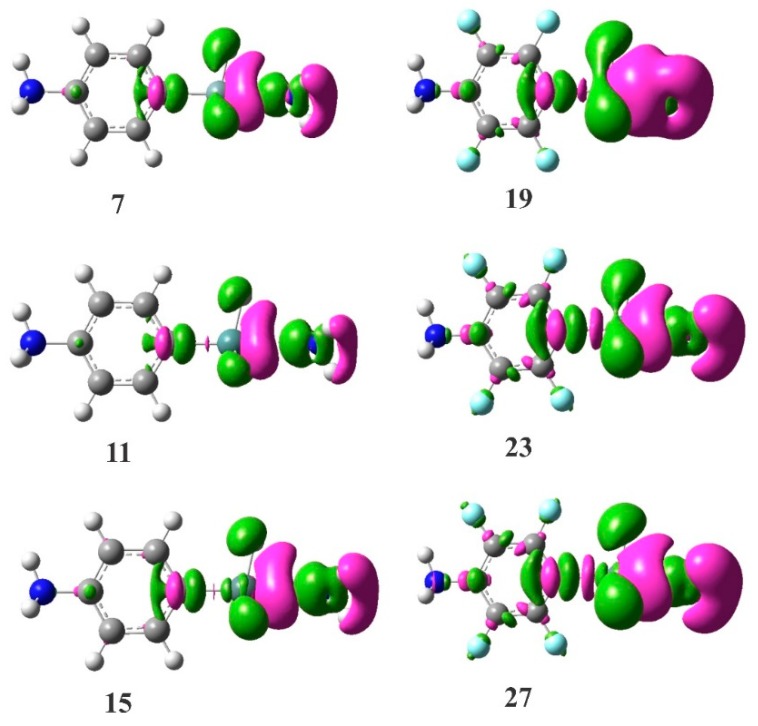
EDD isosurfaces (±0.001 au) of some representative cationic tetrel-bonded complexes. The violet and green regions indicate regions of decreased and increased electron densities, respectively.

Finally, we would like to highlight the electron density redistribution within and between the monomers upon the formation of the cationic tetrel-bonded complexes. Figure 6 shows the EDD plots of some selected complexes. As is evident, the formation of tetrel-bond interaction in these complexes makes a large electron density accumulation region over the nitrogen atom of the Lewis base. The degree of the accumulation depends on the strength of the tetrel-bond, and increases in the order of **15** > **11** > **7** and **27** > **23** > **19**. In contrast, the lone-pair orbital of the nitrogen atom induces an electron density loss area on the M atom. Note that such mutual polarization between the interacting monomers is almost similar as that in other studied tetrel-bonded systems [38,90,93].

### 3.3. Experimental Evidencefor Charge-Assisted Tetrel-Bonds

**Figure 7:**
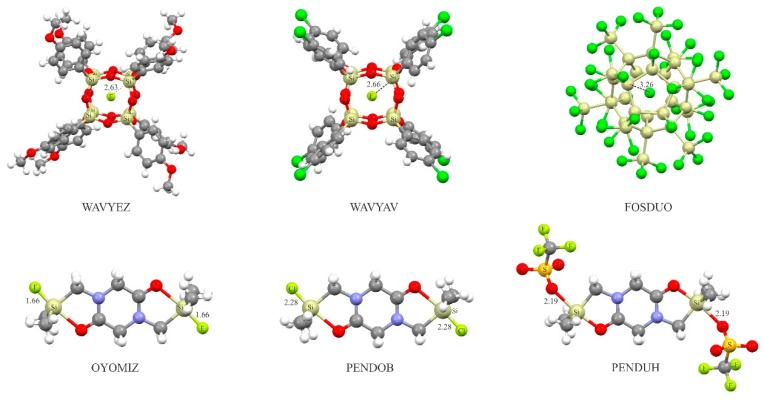
Crystalline structure ofsome selectedanionic tetrel-bonded complexes.

It is noteworthy that the existence of charge-assisted tetrel-bonds described theoretically here may also be confirmed experimentally. To this end, the Cambridge Structure Database (CSD) [99] was examined to analyze whether anionic or cationic tetrel-bonding could be a generally occurring interaction within crystal structures (CSD, version 5.34, November 2012, including three updates). In Figure 7, we show some selected examples and the CSD reference codes of crystal structures in which the anionic tetrel-bonding interaction is observed. In all these complexes, the tetrel-bonding is highly directional, as evidenced by R-M···A^−^ bonding angles close to 180° (R is the electron-withdrawing atom or group attached to the M atom). A quite interesting experimental finding that reports the existence of anionic tetrel-bonding interactions is the formation of crystalline spherosilicate structures, where a fluoride ion is perfectly centered within the octasilsesquioxane cage [100,101]. The X-ray structures are indicated in Figure 7 (WAVYEZ and WAVYAV), where tetrel-bond interactions are confirmed by relatively short contacts between the Si atom and F^−^ anion, which are significantly shorter than the sum of the corresponding vdW radii. Likewise, there also exist attractive anionic tetrel-bonding interactions between the Si atoms and encapsulated Cl^−^ anion in FOSDUO. In Figure 7, we also show the crystalline structures of three binuclear pentacoordinate silicon complexes of diketopiperazine, which gives evidence for the covalent-bonding between the Si atom and the F^−^, Cl^−^ and OSO_2_BF_3_^−^ anions [102]. Note that the shorter Si···F^−^ bond distances compared to the Si···Cl^−^ confirm our earlier finding that the F^−^ has a larger tendency to interact with the tetrel atom than the Cl^−^.

**Figure 8:**
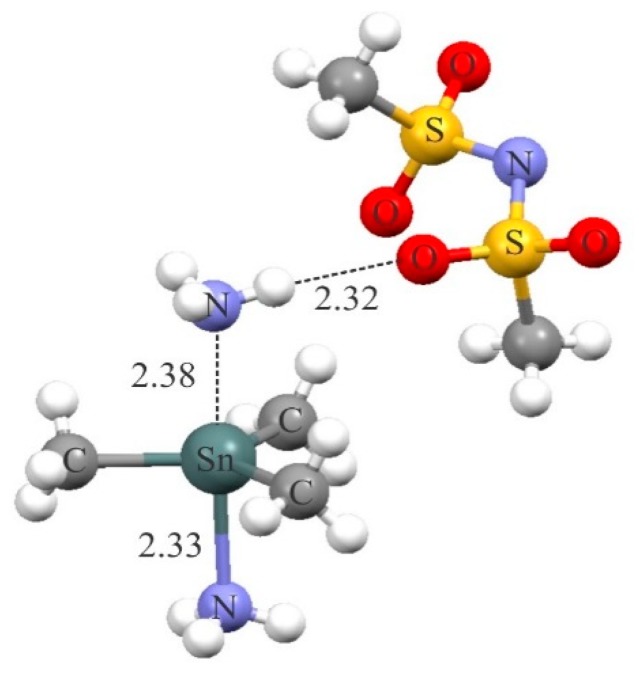
Crystalline structure of the [Sn(Me)_3_(NH_3_)_2_][N(SO_2_Me)_2_] complex.

The existence of short tetrel-bond interaction between the cationic tetrel atom and a potential nitrogen base has been already suggested for the complex [Sn(Me)_3_(NH_3_)_2_][N(SO_2_Me)_2_] [103] (Figure 8). Here, the positively charged Sn(Me)_3_NH_3_ moiety forms a strong Sn···N interaction with the NH_3_ molecule. Meanwhile, there is a short H-bonding interaction between the latter NH_3_ molecule and the negatively charged N(SO_2_Me)_2_ moiety in this complex. The formation of this H-bonding interaction is able to greatly modulate the strength and properties of the tetrel-bonding, as evidenced by the previous theoretical study about the cooperativity effects between the tetrel-bonding and H-bonding interactions [59].

## 4. Conclusions

Using the ab initio calculations, the geometry, interaction energy and bonding properties of anionic and cationic tetrel-bonded complexes were investigated. Our results indicated that these interactions are highly directional due to the localization of a positive electrostatic potential on the tetrel atom and might serve as a molecular linker in supramolecular assemblies. The strength of these charge-assisted tetrel-bonds increases with the increase of the atomic number of the Lewis acid center (Si < Ge < Sn). The QTAIM and NBO approaches were used to deepen the understanding of the nature of the charge-assisted tetrel-bonds. The formation of the anionic and cationic tetrel-bonds results in a sizable electron density redistribution over the interacting subunits, and an increase of the polarization of M-X or M-C bond. In particular, the M atom in very strong tetrel-bonded complexes XF_3_M:A^−^ is characterized by pentavalency, i.e., is hypervalent. Moreover, the application of such charge-assisted tetrel-bonds in crystal materials were characterized and evidenced by a CSD search. The results of this study may provide some new insights into the role of tetrel-bonding interactions in crystalline structure and supramolecular chemistry.

## Figures and Tables

**Table 1:** Binding distances (R_int_, Å), X-M-F angles (θ, °), M-X bond lengths (R_M-X_, Å) and their changes with respect to those of isolated MF_3_X monomers (ΔR_M-X_, Å), interaction energies (E_int_, kcal/mol) of the anionic tetrel-bonded complexes, and the calculated local MEP minimum values (V_S,min_, kcal/mol) of the anions.

Lewis Acid	Anion	R_int_	θ	R_M-X_	ΔR_M-X_	E_int_	V_S,min_
SiF_4_	F^−^	1.679	90.0	1.679	0.105	−70.11	−175.0
	Cl^−^	2.102	91.7	1.665	0.091	−25.00	−141.2
	Br^−^	2.226	92.8	1.657	0.083	−16.35	−132.9
	NC^−^	1.888	92.0	1.661	0.087	−35.03	−141.9
	CN^−^	1.969	91.4	1.663	0.089	−38.86	−135.9
	N_3_^−^	1.904	91.7	1.661	0.087	−30.08	−133.5
SiF_3_CN	F^−^	1.662	91.4	1.970	0.145	−84.37	−175.0
	Cl^−^	2.112	89.9	1.951	0.126	−37.29	−141.2
	Br^−^	2.166	90.6	1.942	0.117	−27.60	−132.9
	NC^−^	1.860	90.5	1.943	0.118	−46.59	−141.9
	CN^−^	1.946	90.0	1.946	0.121	−50.72	−135.9
	N_3_^−^	1.868	89.9	1.945	0.12	−41.87	−133.5
GeF_4_	F^−^	1.773	90.0	1.773	0.085	−79.17	−175.0
	Cl^−^	2.263	90.3	1.764	0.076	−41.26	−141.2
	Br^−^	2.436	90.7	1.760	0.072	−32.99	−132.9
	NC^−^	1.932	91.3	1.762	0.074	−46.62	−141.9
	CN^−^	1.994	90.5	1.762	0.074	−53.57	−135.9
	N_3_^−^	1.940	90.7	1.762	0.074	−43.30	−133.5
GeF_3_CN	F^−^	1.762	90.5	1.994	0.118	−84.92	−175.0
	Cl^−^	2.239	89.7	1.983	0.107	−46.32	−141.2
	Br^−^	2.407	90.0	1.977	0.101	−37.73	−132.9
	NC^−^	1.915	90.8	1.981	0.105	−50.96	−141.9
	CN^−^	1.979	90.0	1.979	0.103	−58.35	−135.9
	N_3_^−^	1.921	90.0	1.981	0.105	−47.94	−133.5
SnF_4_	F^−^	1.930	90.0	1.930	0.048	−93.58	−175.0
	Cl^−^	2.371	89.5	1.936	0.054	−61.84	−141.2
	Br^−^	2.523	90.4	1.934	0.052	−54.44	−132.9
	NC^−^	2.078	90.1	1.933	0.051	−62.50	−141.9
	CN^−^	2.144	90.1	1.932	0.05	−70.26	−135.9
	N_3_^−^	2.083	90.3	1.932	0.05	−60.07	−133.5
SnF_3_CN	F^−^	2.144	90.1	2.144	0.091	−96.30	−175.0
	Cl^−^	2.357	90.8	2.141	0.088	−64.11	−141.2
	Br^−^	2.508	89.2	2.139	0.086	−56.59	−132.9
	NC^−^	2.067	90.9	2.134	0.081	−64.21	−141.9
	CN^−^	2.132	90.0	2.132	0.079	−72.26	−135.9
	N_3_^−^	2.073	90.1	2.133	0.080	−62.09	−133.5

**Table 2:** Electron density (*ρ*_BCP_, au), its Laplacian (∇^2^*ρ*_BCP_, au) and total electron energy density (H_BCP_, au) at the M···A^−^ BCPs, and NBO stabilization energy (E^(2)^, kcal/mol), atomic charge on the M atom (q_M_, e), net charge-transfer (q_CT_, e) and Wiberg bond index (WBI) values of the anionic tetrel-bonded complexes.

Lewis Acid	Anion	*ρ*_BCP_	∇^2^*ρ*_BCP_	H_BCP_	E^(2)^	q_M_	q_CT_	WBI
SiF_4_	F^−^	0.112	0.879	−0.025	53.63	2.64	0.26	0.48
	Cl^−^	0.065	0.152	−0.030	38.72	2.54	0.33	0.51
	Br^−^	0.054	0.060	−0.028	32.06	2.55	0.31	0.49
	NC^−^	0.085	0.421	−0.029	69.31	2.63	0.21	0.37
	CN^−^	0.081	0.280	−0.045	47.92	2.50	0.34	0.56
	N_3_^−^	0.086	0.368	−0.034	45.64	2.42	0.59	0.41
SiF_3_CN	F^−^	0.117	0.936	−0.028	65.86	2.50	0.27	0.56
	Cl^−^	0.070	0.183	−0.035	56.59	2.34	0.39	0.60
	Br^−^	0.064	0.080	−0.034	49.40	2.32	0.40	0.60
	NC^−^	0.093	0.301	−0.049	89.12	2.45	0.25	0.60
	CN^−^	0.086	0.299	−0.049	61.17	2.30	0.39	0.60
	N_3_^−^	0.094	0.415	−0.040	60.75	2.42	0.58	0.46
GeF_4_	F^−^	0.130	0.774	−0.050	54.69	2.68	0.27	0.42
	Cl^−^	0.084	0.130	−0.038	51.64	2.52	0.38	0.57
	Br^−^	0.073	0.055	−0.032	46.01	2.50	0.40	0.58
	NC^−^	0.107	0.364	−0.048	78.64	2.66	0.22	0.38
	CN^−^	0.112	0.191	−0.058	55.20	2.51	0.38	0.58
	N_3_^−^	0.109	0.308	−0.053	53.60	2.60	0.58	0.44
GeF_3_CN	F^−^	0.134	0.800	−0.053	58.67	2.51	0.27	0.44
	Cl^−^	0.089	0.135	−0.042	60.69	2.31	0.42	0.63
	Br^−^	0.078	0.053	−0.036	55.20	2.28	0.46	0.65
	NC^−^	0.112	0.381	−0.052	91.00	2.45	0.25	0.42
	CN^−^	0.116	0.194	−0.062	61.82	2.29	0.42	0.63
	N_3_^−^	0.115	0.323	−0.058	59.72	2.40	0.58	0.48
SnF_4_	F^−^	0.113	0.687	−0.029	34.37	2.93	0.21	0.36
	Cl^−^	0.081	0.195	−0.027	42.18	2.75	0.36	0.37
	Br^−^	0.072	0.117	−0.024	40.60	2.71	0.40	0.60
	NC^−^	0.098	0.378	−0.031	53.92	2.90	0.19	0.34
	CN^−^	0.094	0.229	−0.038	37.04	2.77	0.33	0.53
	N_3_^−^	0.099	0.384	−0.066	34.80	2.84	0.27	0.40
SnF_3_CN	F^−^	0.101	0.702	−0.031	33.81	2.77	0.22	0.38
	Cl^−^	0.084	0.199	−0.029	44.15	2.55	0.40	0.61
	Br^−^	0.075	0.118	−0.026	42.58	2.50	0.45	0.66
	NC^−^	0.101	0.385	−0.034	53.18	2.71	0.22	0.37
	CN^−^	0.098	0.230	−0.040	34.38	2.55	0.37	0.58
	N_3_^−^	0.098	0.341	−0.037	31.10	2.65	0.54	0.44

**Table 3:** Binding distances (R_int_, Å), C-M-H angles (θ, °) and interaction energies (E_int_, kcal/mol) of the cationic tetrel-bonded complexes.

Complex		R_int_	θ	E_int_
**7**	1+NH_3_	2.567	101.1	−7.69
**8**	1+NH_2_OH	2.459	101.4	−9.25
**9**	1+NH_2_CH_3_	2.291	98.2	−13.58
**10**	1+NH_2_NH_2_	2.245	97.5	−15.05
**11**	2+NH_3_	2.790	102.6	−9.82
**12**	2+NH_2_OH	2.724	103.0	−11.86
**13**	2+NH_2_CH_3_	2.538	100.2	−17.44
**14**	2+NH_2_NH_2_	2.486	99.9	−20.8
**15**	3+NH_3_	2.735	100.5	−13.36
**16**	3+NH_2_OH	2.671	100.2	−16.65
**17**	3+NH_2_CH_3_	2.568	97.7	−19.11
**18**	3+NH_2_NH_2_	2.548	98.1	−22.46
**19**	4+NH_3_	2.330	99.4	−10.89
**20**	4+NH_2_OH	2.277	99.0	−13.47
**21**	4+NH_2_CH_3_	2.195	96.7	−17.42
**22**	4+NH_2_NH_2_	2.175	97.0	−20.86
**23**	5+NH_3_	2.527	100.3	−14.25
**24**	5+NH_2_OH	2.498	101.1	−18.48
**25**	5+NH_2_CH_3_	2.370	98.4	−22.55
**26**	5+NH_2_NH_2_	2.352	98.6	−25.5
**27**	6+NH_3_	2.586	99.0	−17.02
**28**	6+NH_2_OH	2.574	100.3	−20.08
**29**	6+NH_2_CH_3_	2.487	98.1	−23.66
**30**	6+NH_2_NH_2_	2.479	98.7	−26.98

**Table 4:** Electron density (*ρ*_BCP_, au), its Laplacian (∇^2^*ρ*_BCP_, au) and total electron energy density (H_BCP_, au) at the M···Z BCPs, and NBO stabilization energies due to the LP(N) →BD*_M-C_ orbital interaction (E^(2)^, kcal/mol), net charge-transfer (q_CT_, e) and Wiberg bond index (WBI) values of the cationic tetrel-bonded complexes.

Complex		*ρ*_BCP_	∇^2^*ρ*_BCP_	H_BCP_	E^(2)^	q_CT_	WBI
**7**	1+NH_3_	0.024	0.049	−0.003	10.42	0.072	0.114
**8**	1+NH_2_OH	0.028	0.066	−0.017	11.20	0.075	0.129
**9**	1+NH_2_CH_3_	0.039	0.084	−0.021	13.45	0.078	0.135
**10**	1+NH_2_NH_2_	0.043	0.111	−0.024	14.52	0.080	0.144
**11**	2+NH_3_	0.029	0.055	−0.005	12.28	0.076	0.134
**12**	2+NH_2_OH	0.034	0.062	−0.019	14.55	0.079	0.155
**13**	2+NH_2_CH_3_	0.044	0.088	−0.026	17.70	0.082	0.167
**14**	2+NH_2_NH_2_	0.048	0.099	−0.030	19.14	0.084	0.176
**15**	3+NH_3_	0.035	0.090	−0.007	15.25	0.079	0.155
**16**	3+NH_2_OH	0.039	0.072	−0.008	17.10	0.081	0.168
**17**	3+NH_2_CH_3_	0.048	0.083	−0.022	18.75	0.084	0.182
**18**	3+NH_2_NH_2_	0.052	0.111	−0.026	20.40	0.086	0.193
**19**	4+NH_3_	0.035	0.053	−0.008	13.28	0.079	0.149
**20**	4+NH_2_OH	0.039	0.090	−0.023	14.48	0.084	0.189
**21**	4+NH_2_CH_3_	0.047	0.136	−0.030	16.05	0.087	0.201
**22**	4+NH_2_NH_2_	0.050	0.141	−0.033	18.60	0.090	0.228
**23**	5+NH_3_	0.043	0.113	−0.030	15.08	0.082	0.165
**24**	5+NH_2_OH	0.046	0.097	−0.021	16.69	0.085	0.172
**25**	5+NH_2_CH_3_	0.054	0.121	−0.028	18.82	0.089	0.191
**26**	5+NH_2_NH_2_	0.059	0.123	−0.032	20.80	0.094	0.206
**27**	6+NH_3_	0.046	0.089	−0.010	17.04	0.092	0.180
**28**	6+NH_2_OH	0.049	0.093	−0.026	18.92	0.095	0.196
**29**	6+NH_2_CH_3_	0.056	0.131	−0.033	19.77	0.096	0.212
**30**	6+NH_2_NH_2_	0.060	0.141	−0.036	22.50	0.098	0.225

## Supplementary Materials

The following are available online. Figure S1: Optimized structure of the anionic tetrel-bonded complexes, Figure S2: Optimized structure of the cationic tetrel-bonded complexes **7**–**30**, Figure S3: Correlation between the stabilization energy, due to the LP(N) → BD*M-C orbital interaction, and interaction energies of cationic tetrel-bonded complexes **7**–**30**, Figure S4: Correlation between the net charge-transfer and interaction energies of cationic tetrel-bonded complexes **7**–**30**.
