# Supplementary material for: Strong Tetrel Bonds: Theoretical Aspects and Experimental Evidence
Source: Molecules. 2018 Oct 15;23(10):2642. doi: 10.3390/molecules23102642 (PMC6222713; doi:10.3390/molecules23102642)

“ Supporting Information”

Strong Tetrel Bonds: Theoretical Aspects and Experimental Evidence

Mehdi D. Esrafili * and Parisasadat Mousavian

Laboratory of Theoretical Chemistry, Department of Chemistry, University of Maragheh, Maragheh, 5513864596, Iran; p.mosavian1327@gmail.com

* Correspondence: esrafili@maragheh.ac.ir; Tel.: +98-42-12237955; Fax: +98-4212276060

Figure S1. Optimized structure of the anionic tetrel-bonded complexes

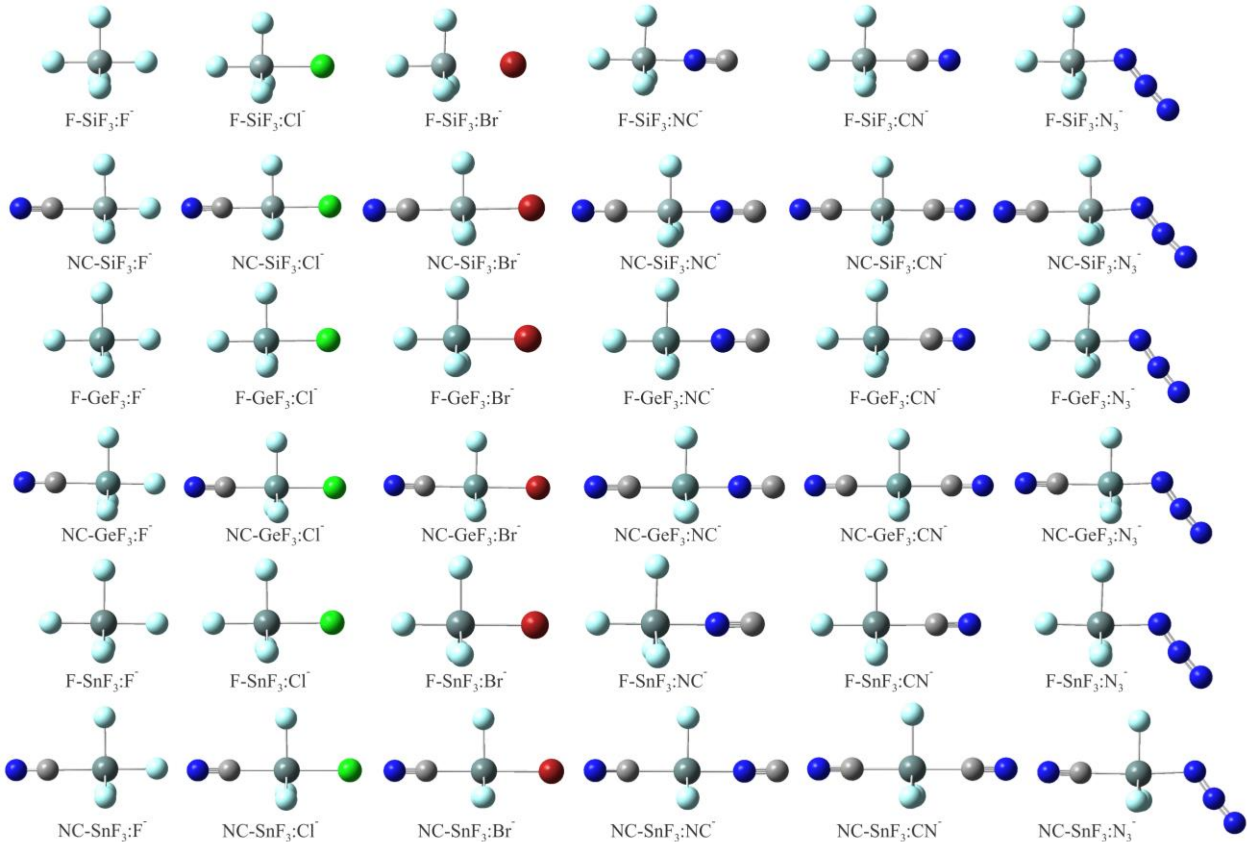

Figure S2. Optimized structure of the cationic tetrel-bonded complexes **7-30**

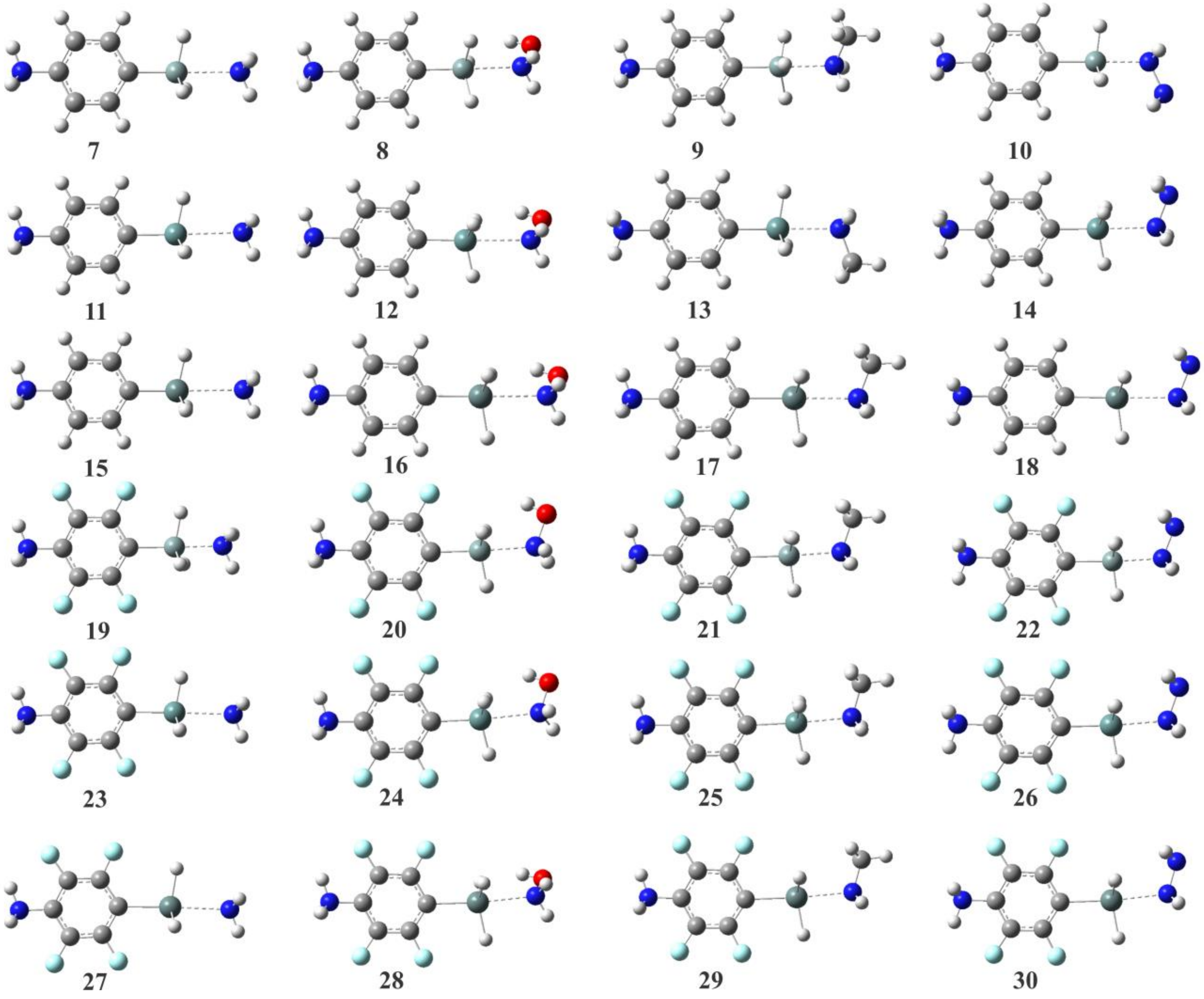

Figure S3. Correlation between the stabilization energy, due to the LP(N) → BD*$_{M-C}$ orbital interaction, and interaction energies of cationic tetrel-bonded complexes **7-30**

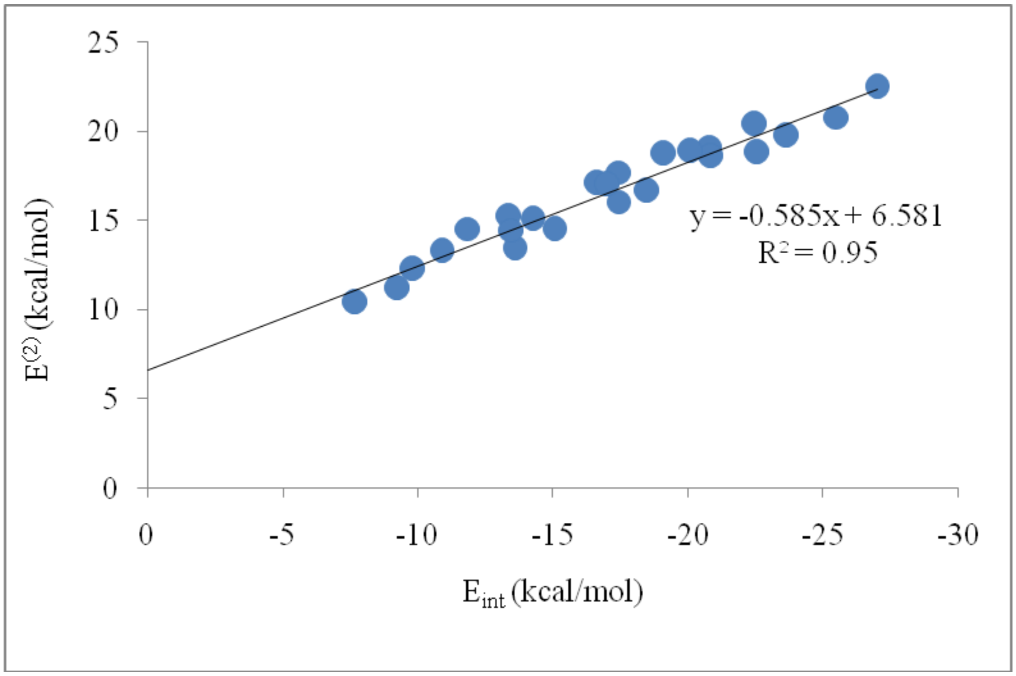

Figure S4. Correlation between the net charge-transfer and interaction energies of cationic tetrel-bonded complexes **7-30**

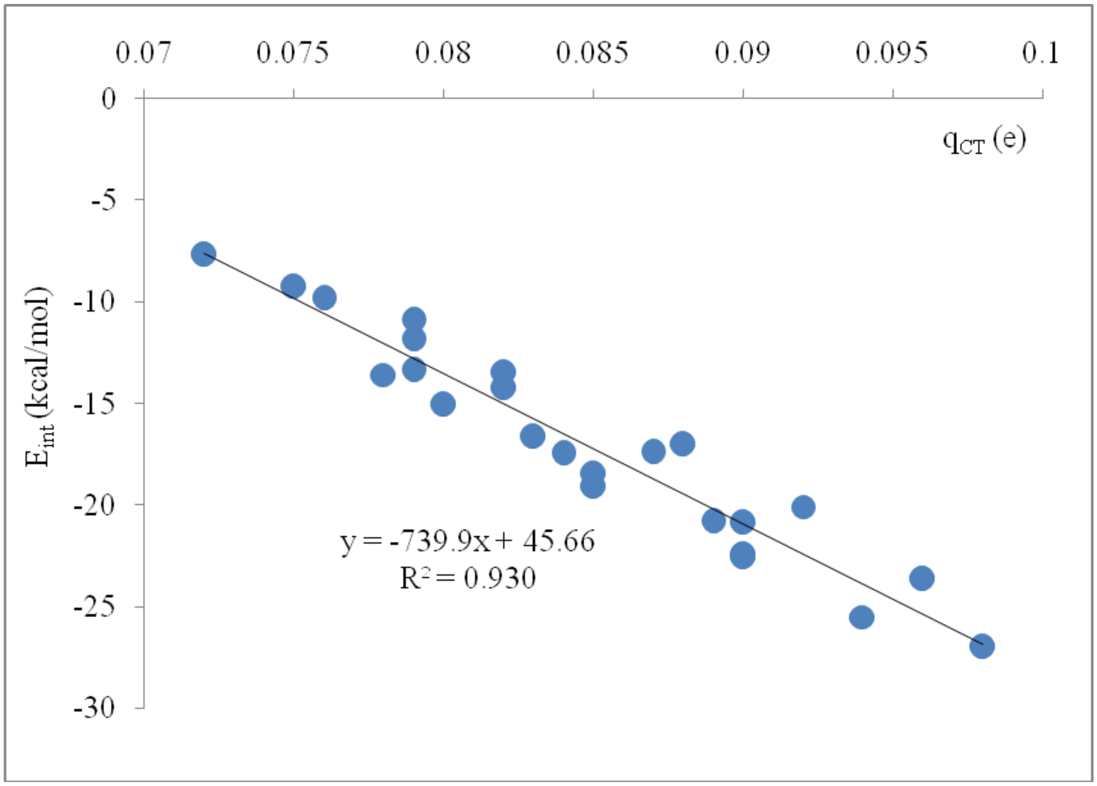

Supplement: Supplementary file 1 [file molecules-23-02642-s001.pdf]
